# APX001 and Other Gwt1 Inhibitor Prodrugs Are Effective in Experimental *Coccidioides immitis* Pneumonia

**DOI:** 10.1128/AAC.01715-18

**Published:** 2019-01-29

**Authors:** Suganya Viriyakosol, Mili Kapoor, Sharon Okamoto, Jonathan Covel, Quinlyn A. Soltow, Michael Trzoss, Karen Joy Shaw, Joshua Fierer

**Affiliations:** a VA Healthcare, San Diego, California, USA; b Amplyx Pharmaceuticals, San Diego, California, USA; c Division of Infectious Diseases, Department of Medicine, UC San Diego School of Medicine, San Diego, California, USA

**Keywords:** 1-aminobenzotriazole, APX001, APX001A, *Coccidioides*, GPI anchor biosynthesis, Gwt1, antifungal therapy

## Abstract

Coccidioidomycosis is a systemic fungal infection caused by the inhalation of the arthroconidia of either of two closely related dimorphic fungi, Coccidioides immitis and C. posadasii, that are endemic in the southwestern United States and other areas in the Western Hemisphere. Chronic cavitary pulmonary infections and extrapulmonary sites of infection are very difficult to treat and often require lifelong azole therapy. APX001A is the first in a new class of broad-spectrum antifungal agents that inhibit Gwt1, an enzyme which is required for cell wall localization of glycosylphosphatidylinositol (GPI)-anchored mannoproteins in fungi. APX001A and several analogs were highly active against clinical isolates of *Coccidioides*, inhibiting hyphal growth at low nanogram/ml concentrations. APX001 is the N-phosphonooxymethyl prodrug of APX001A, currently in clinical trials for the treatment of invasive fungal infections. Mice were treated orally once daily with 26 mg/kg/day of APX001 and the prodrug analog APX2097, 2 h after administration of the pan-cytochrome P450 inhibitor 1-aminobenzotriazole, which was used to enhance drug half-life and exposures to more closely mimic human pharmacokinetics of APX001A. Five days of treatment reduced lung colony counts by nearly 3 logs and prevented dissemination, similar to the efficacy of fluconazole dosed orally at 25 mg/kg twice daily. In a survival experiment, both APX001- and APX2097-treated mice survived significantly longer than control and fluconazole-treated mice. APX001 and other members of this new class of antifungal agents may offer great promise as effective therapies for coccidioidomycosis.

## Abstract (precis)

Coccidioidomycosis is a systemic fungal infection caused by the inhalation of the arthroconidia of either of two closely related dimorphic fungi, Coccidioides immitis and C. posadasii, that are endemic in the southwestern United States and other areas in the Western Hemisphere. Chronic cavitary pulmonary infections and extrapulmonary sites of infection are very difficult to treat and often require lifelong azole therapy.

## INTRODUCTION

Coccidioidomycosis (San Joaquin Valley Fever) is a systemic fungal infection that is endemic in the Southwestern United States from West Texas to Southern and Central California and in arid regions in Central and South America (1). The disease is caused by two closely related species, Coccidioides immitis and C. posadasii (2), both of which are dimorphic. Desert rodents (the natural host [3]) and humans become infected by inhaling arthroconidia (spores) that are aerosolized by wind. After the spores enter a mammalian host, they convert to round cells that enlarge to become spherules. The spherules are large, spherical structures that grow to a diameter of >100 μm and reproduce by segmenting internally into hundreds of endospores that are released when the spherule ruptures. In the United States, coccidioidomycosis is a reportable infection only in California and Arizona. The incidence in those two states has been increasing in recent years (4). Many infections are either asymptomatic or so mild that people do not seek medical attention. However, symptomatic pneumonia can be severe and debilitating; ∼5% of infections spread to extrapulmonary sites and are extremely difficult to treat. Disseminated infection accounts for most of the deaths due to coccidioidomycosis (5).

The first effective treatment approved in the United States for coccidioidomycosis was amphotericin B deoxycholate, which is quite toxic (6). However, even the newer lipid formulations demonstrate toxicity. Although ketoconazole is approved by the U.S. Food and Drug Administration for the treatment of coccidioidomycosis, it is no longer recommended for treatment of coccidioidomycosis due to toxicity and lesser potency than the newer triazoles (7). Fluconazole and other triazoles are now the most frequently used drugs to treat coccidioidomycosis; however, relapse of coccidioidomycosis is common when they are discontinued (7). The benefits of fluconazole and itraconazole in chronic infections are not dramatic, requiring a complicated scoring system developed by the Mycoses Study Group to show a beneficial effect (8, 9). In addition, there is recent evidence that some clinical isolates of *Coccidioides* have high MIC values for fluconazole (9). Thus, there is a need for new drugs for this infection.

In this study, we evaluated the *in vitro* and *in vivo* activity of a novel class of broad-spectrum antifungal agents against *Coccidioides* spp. These compounds are structurally and mechanistically unrelated to other antifungal drugs and inhibit the highly conserved fungal enzyme Gwt1, which is required for cell wall localization of glycosylphosphatidylinositol (GPI)-anchored mannoproteins in fungi (10–12). In Candida albicans, these GPI-anchored mannoproteins are often components of the cell wall, they are surfaced exposed, and they have other diverse cellular functions (12, 13).

**FIG 1:**
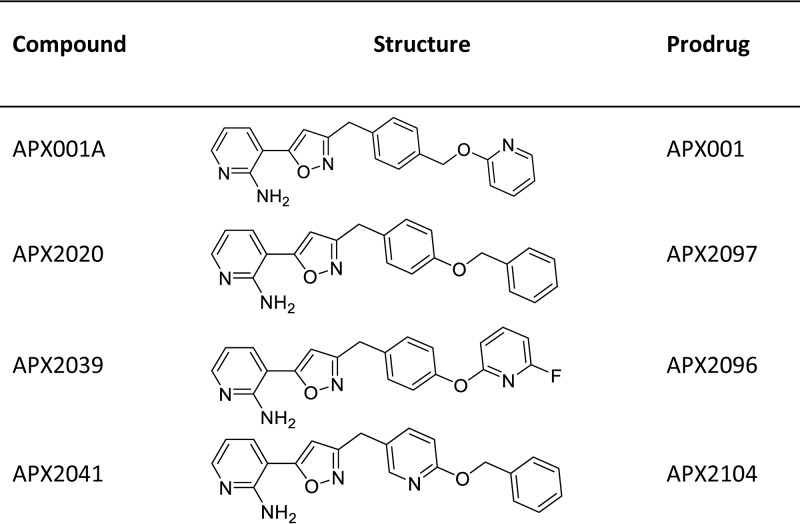
Structures of Gwt1 inhibitors.

For assessment of *in vivo* efficacy, *N*-phosphonooxymethyl prodrugs of these molecules (Fig. 1) were synthesized in an analogous method to the synthesis of APX001 (14, 15). These prodrugs are rapidly and completely metabolized by host alkaline phosphatases to the active moieties (16–18). APX001A has a short half-life in mice (1.4 to 2.5 h) after administration of the prodrug APX001 (18), whereas phase 1 studies in healthy volunteers have shown a half-life of 2.5 days and exposures of ≥200 μg ⋅ h/ml (19, 20). To enable dosing regimens that more closely mimic human pharmacokinetics, we orally administered 1-aminobenzotriazole (ABT), a nonselective suicide inhibitor of cytochrome P450 (CYP) enzymes (21), 2 h prior to the oral administration of APX prodrugs. Previous studies have shown that ABT extends the half-life and increases the exposure of APX001A and other related APX molecules, after administration of the corresponding prodrugs (17, 22). ABT has been shown to have no *in vitro* antifungal activity against four species (Candida albicans, Cryptococcus neoformans, Aspergillus fumigatus, and Scedosporium apiospermum) when tested at concentrations up to 250 μg/ml, nor does it demonstrate synergistic effects when evaluated in combination with APX001A (M. Kapoor, unpublished observations).

## RESULTS

### *In vitro* activity of Gwt1 inhibitors versus *Coccidioides* spp.

The *in vitro* activity of the active moiety APX001A was evaluated against three laboratory strains of *Coccidioides* (Table 1). Since there is no standardized CLSI method for *Coccidioides*, we compared the minimal effective concentration (MEC) causing abnormal hyphal growth (short abundant branching) in a broth microdilution assay and also determined the MIC values of APX001A, fluconazole, posaconazole, and amphotericin B against *Coccidioides* arthroconidia using a microbroth serial dilution assay. The MEC value for APX001A was approximately 1 to 3 logs lower than the MIC value and was easier to determine precisely with no interobserver variation (Table 1). The use of a MEC endpoint for APX001A and the echinocandins has been established for other molds, including *Aspergillus* species (23–25). The MIC values for posaconazole ranged between 0.03 to 0.125 μg/ml and >16 μg/ml for fluconazole, when read at the more stringent endpoint of 100% inhibition rather than the less-stringent CLSI reading of 50% inhibition for azoles and other molds (26).

**TABLE 1:** *In vitro* susceptibility profiles

Strain	MEC (μg/ml), APX001A	MIC (μg/ml)^*a*^			
		APX001A	FLC	AMB	POS
C. immitis RS	0.002–0.004	8	>16	0.125	0.06–0.125
C. posadasii C735	0.004	0.03	>16	0.25	0.06–0.125
C. posadasii Silvera	0.008	8	>16	0.25	0.03

a The MIC value was read at 100% inhibition. FLC, fluconazole; AMB, amphotericin B; POS, posaconazole.

The activity of 33 APX001A analogs were evaluated against one strain each of *C. immitis* and C. posadasii. Sixteen compounds were active at levels ≤0.016 μg/ml (data not shown), and two of the most active compounds (APX2020 and APX2041) were chosen for further analysis against a larger panel of strains that included five isolates each of C. posadasii and *C. immitis* (Fig. 1, Table 2). The activity of these compounds was compared to APX001A and posaconazole, one of the most potent azoles against *Coccidioides* (9). All three Gwt1 inhibitors were highly active, with MEC_90_ values of 0.002, 0.004, and 0.008 μg/ml for APX2041, APX2020, and APX001A, respectively, while the MIC_90_ for posaconazole was 0.125 μg/ml (Table 2). The ranges of MEC values for *C. immitis* appeared to be slightly lower (2- to 8-fold) than those for C. posadasii for the three Gwt1 inhibitors (Table 2).

**TABLE 2:** Activity of Gwt1 inhibitors versus C. immitis and C. posadasii

Strain or parameter	Source	MEC (μg/ml)			MIC (μg/ml)
		APX001A	APX2020	APX2041	POS
C. immitis					
RS	Lab	0.002–0.004^*a*^	0.002–0.004	0.002–0.004	0.06–0.125
B2358	CDC	0.004	0.004	0.000125	0.016
F40	Clinical	0.004	0.002	0.001	0.125
F1	Clinical	0.002	0.001	0.001	0.125
UCSD2	Clinical	0.001	0.001	0.00025	0.125
					
C. posadasii					
F6	Clinical	0.016	0.004	0.001	0.125
Silvera	Lab	0.008	0.008	0.004	0.03
F5	Clinical	0.008	0.004	0.001	0.016
C735	Lab	0.004	0.002	0.002	0.06–0.125
D2A	Clinical	0.004	0.002	0.001	0.03
					
Parameters					
GM		0.004	0.002	0.001	0.054
MEC_90_/MIC_90_		0.008	0.004	0.002	0.125

a The lower value of the susceptibility range was used to calculate the geometric mean (GM) and 90% minimal effective concentration (MEC_90_).

**FIG 2:**
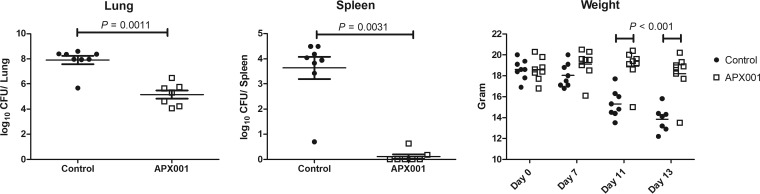
Efficacy of APX001 in a murine model of coccidioidomycosis. Mice were infected intranasally with *C. immitis* RS arthroconidia and 50 mg/kg of APX001 was administered twice daily for 5 days beginning 7 days postinfection. Mice were sacrificed on day 13, 1 day after the last day of treatment, and colony counts were assessed from the lung and spleen. Each symbol represents one mouse. The horizontal lines show the geometric mean and SEM for the lung and spleen colony counts (CFU). Horizontal lines in the weight panel correspond to the calculated mean weight. The differences in mean weight of treated and control mice on days 11 and 13 were analyzed by two-way ANOVA (GraphPad Prism) and were highly significant (*P* < 0.001).

***In vivo* activity of Gwt1 inhibitors versus *C. immitis*. (i) Activity of APX001 in a pulmonary murine coccidioidomycosis model.** A mouse model of coccidioidomycosis was used to evaluate the activity of APX001 against the pathogenic form of the fungus. B6 mice were chosen due to their genetic susceptibility to this infection (27). Thus, this model would be analogous to treating patients who are genetically predisposed to disseminated infection, the most challenging group of patients to treat. Mice were infected by inhalation of ∼200 arthroconidia/mouse, and treatment was initiated 7 days later in order to allow enough time for the arthroconidia to transform into spherules. Mice were then treated twice daily by oral gavage with 50 mg/kg of APX001 (equivalent to 77 mg/kg/day of the active moiety APX001A, using a conversion factor of 1.3 to account for the methyl phosphate group) for 5 consecutive days. The geometric mean log_10_ CFU/g in the lungs and spleens in the untreated control groups were 7.91 and 3.99, respectively (Fig. 2). APX001 treatment reduced the lung CFU geometric mean lung CFU by nearly 2.75 logs (*P* = 0.0011) and prevented dissemination to the spleen (*P* = 0.0031). Brain CFU were also examined, and all eight APX001-treated animals demonstrated complete sterilization versus <5 CFU/g of brain tissue in controls (*P* = 0.0002; data not shown). As further evidence for the efficacy of APX001 treatment, mice treated with APX001 did not lose weight, whereas the control mice lost 24% of body weight by day 13 (*P* < 0.001) (Fig. 2).

**(ii) ABT has no antifungal activity in mice.** Due to the short half-life of APX001A after APX001 administration in mice (1.4 to 2.5 h) and the importance of area under the curve (AUC)/MIC as the driver of efficacy (18), we concluded that twice-daily (BID) dosing was not an optimal treatment regimen for coccidioidomycosis. To more closely mimic the long half-life (2 to 2.5 days) observed in phase 1 clinical studies (19, 20), we evaluated the use of the pan-CYP450 inhibitor ABT in the coccidioidomycosis model. ABT had been previously shown to extend the half-life and increase the AUC of the four Gwt1 inhibitors shown in Fig. 1 by 8.6- to 15-fold after dosing of the prodrug (17, 22).

**FIG 3:**
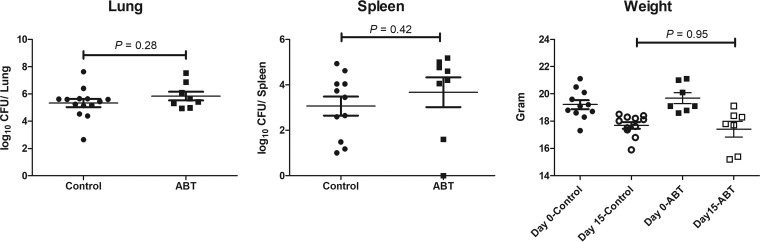
ABT alone has no antifungal effect in mice. Infected mice were treated with a single daily dose of ABT for 5 days and sacrificed on day 13, the day after the last ABT dose. Fungal colony counts were log transformed. Geometric mean CFU/organ values ± the SEM were calculated and compared using an unpaired *t* test (Prism, v7.01). The mean weights ± the SEM were calculated, and there was no significant difference in the weights of untreated and ABT-treated mice on day 15 after infection.

To determine whether ABT had an antifungal or toxic effect in this model, mice were infected with ∼200 arthroconidia/mouse and single daily doses of 50 mg/kg ABT were administered starting 4 days after infection and continuing for 5 days. The data in Fig. 3 show that log_10_ CFU/lung and spleen were not significantly different from the untreated control group (*P* > 0.2 for both), demonstrating no antifungal effect of ABT. In addition, the administration of ABT to infected mice did not significantly decrease body weight versus the vehicle control (*P* = 0.95) (Fig. 3) or cause an increase in serum alanine transaminase (ALT) or serum bilirubin (data not shown).

**FIG 4:**
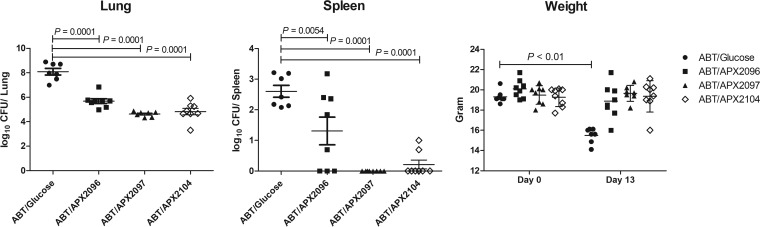
Reduction in fungal burden upon treatment with three Gwt1 prodrugs in a mouse model of pulmonary coccidioidomycosis. Mice were infected and treated as in Fig. 2, except that mice were pretreated with 50 mg/kg ABT by oral gavage 2 h prior to the administration of APX prodrugs or buffer starting 7 days after infection. Mice were weighed at the start and conclusion of the experiment and were sacrificed 1 day after their last dose. After log_10_ transformation, values for the geometric mean CFU/organ ± the SEM were calculated and compared using an unpaired *t* test (Prism, v7.01). If there were >2 groups, the differences in the means of treated and control groups were compared using Dunnett’s ANOVA test. A *P* value of ≤0.05 was considered statistically significant.

**(iii) Efficacy of three Gwt1 inhibitor prodrugs in the treatment of pulmonary coccidioidomycosis: evaluation of CFU.** The activities of three APX001 analogs were evaluated in the coccidioidomycosis mouse model. These compounds included the *N*-phosphonooxymethyl prodrugs of APX2020 and APX2041, along with a third molecule APX2039 (Fig. 1). Although APX2039 was 2- to 4-fold less active against the *C. immitis* RS strain used in the mouse model (MEC = 0.008 μg/ml), the prodrug APX2096 had previously been shown to have improved pharmacokinetics and better efficacy in a cryptococcal meningitis model of infection (17). Mice were infected as in Fig. 2 with ∼200 arthroconidia/mouse, and treatment was initiated on day 7 after infection as before, but in this experiment the mice were pretreated with 50 mg/kg ABT by oral gavage 2 h prior to administration of 26 mg/kg APX prodrugs by oral gavage. This treatment regimen was continued for 5 days. Mice were weighed at the start and conclusion of the experiment and sacrificed 1 day after the last dose. The reduction in fungal colony counts (CFU) in the lung and spleen upon treatment with the three respective prodrugs APX2097, APX2104, and APX2096 (Fig. 1) is shown in Fig. 4. Efficacy was observed for all three treatments compared to the control plus ABT, as measured by significant decreases in log_10_ CFU organ (lung, *P* = 0.0001; spleen, *P* < 0.01). Only the control mice lost weight and at the end of treatment they weighed significantly less than the treated mice (*P* < 0.01). However, APX2096 did not reduce dissemination to the spleen as effectively as the other two derivatives and was thus not pursued further (Fig. 4).

**FIG 5:**
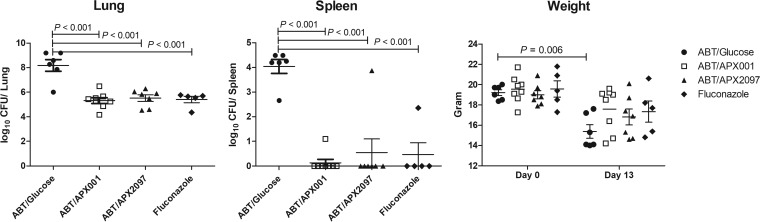
Reduction in fungal burden upon treatment with APX001 and APX2097 in comparison to fluconazole. Mice were infected and treated with the ABT and APX prodrugs as in Fig. 3. Fluconazole was administered orally twice daily. Values for the geometric mean CFU/organ ± the SEM were calculated and compared using a paired *t* test (Prism, v7.01). If there were >2 groups, the differences in the means of treated and control groups were compared using Dunnett’s ANOVA test. All of the treatment groups had significant lower colony counts than the untreated control in lungs and spleen (*P* < 0.001). Only the untreated mice had a statistically significant eight loss on day 13 after infection compared to their starting weight (*P* = 0.006).

**FIG 6:**
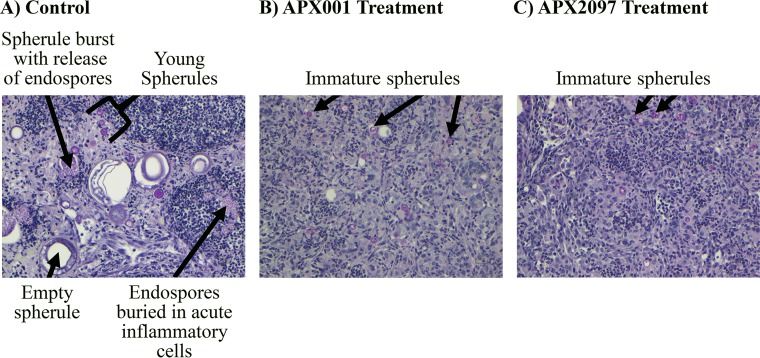
Histological analysis of lung tissue sections in control versus APX001- and APX2097-treated mice. Mice were infected with *C. immitis* RS as described in Materials and Methods and then treated once daily with 50 mg/kg ABT plus APX001 or APX2097 at 26 mg/kg for 5 days. Control mice received only ABT. Lungs were removed a few hours after the last dose, fixed in glutaraldehyde, and then stained with PAS prior to microscopic examination (×20 magnification). (A) The control lungs showed many spherules in all stages of development and a myriad of endospores from ruptured spherules, surrounded by acute and chronic inflammatory cells. (B) APX001-treated mice had many small, immature spherules that were primarily inside macrophages. There were no fully grown spherules and few if any endospores. (C) The lungs of APX2097-treated mice had an appearance similar to the lungs of APX001-treated mice.

We next compared the *in vivo* activities of once daily APX001 and APX2097 to the activity of fluconazole. Fluconazole (25 mg/kg), which is considered first-line therapy in the treatment of coccidioidomycosis in humans (7), was administered orally BID by gavage without ABT pretreatment. Mice were sacrificed 1 day after they had received treatment for 5 days for assessment of the CFU/g of tissue. All three treatment groups had significantly lower CFU/lung than the control group, and all prevented dissemination to the spleen (with the exception of one mouse in each group) (Fig. 5). We repeated this experiment (excluding the fluconazole group) to evaluate the appearance of the spherules in the infected lungs. Figure 6 shows representative lung fields mice treated with ABT/glucose, APX001, and APX2097. The lung from the control mouse shows spherules in all stages of maturation and numerous free endospores, while the spherules in the APX001- and APX2097-treated mice were all small and immature, and many had been ingested by macrophages.

**FIG 7:**
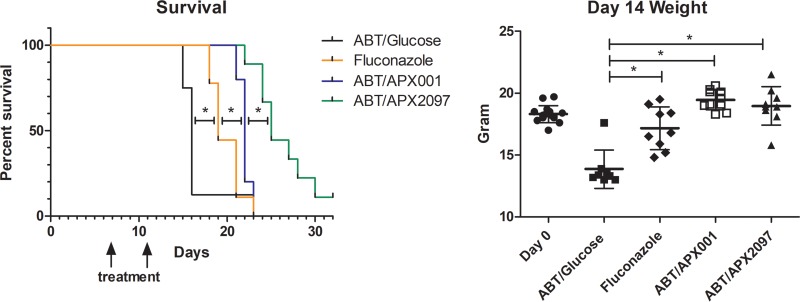
Comparison of Kaplan-Meir survival curves and end-of-treatment weights of mice treated with APX001, APX2097, or fluconazole compared to untreated controls. Mice were infected and treated as described in Fig. 3. The arrows show the days of treatment. Kaplan-Meir survival curves were compared by log rank analysis (Prism, v7.01). All three treatment groups survived significantly longer than the control mice. Differences between the three treatment groups was also significant. The mean body weights of the three treatment groups and the untreated control on day 14 posttreatment were compared by ANOVA (Tukey’s multiple-comparison test; Prism, v7.01). There were no significant differences in the weights of fluconazole-, APX001-, and APX2097-treated mice. *, *P* < 0.01 for both graphs.

**(iv) Efficacy of three Gwt1 inhibitor prodrugs in the treatment of pulmonary coccidioidomycosis: evaluation of survival.** The same infection and dosing conditions were utilized as shown in Fig. 5; however, the endpoint was survival 30 days after infection (18 days after the last treatment dose). As shown in Fig. 7, the fluconazole-treated mice survived significantly longer than the control mice (*P* < 0.01). However, mice treated with APX001 survived significantly longer than the fluconazole-treated mice (*P* < 0.01), and the mice treated with APX2097 survived significantly longer than the APX001-treated mice (*P* < 0.01). The one surviving mouse in the APX2097 group at the end of the experiment was infected.

## DISCUSSION

In this study, we demonstrated that oral administration of the prodrug APX001 and three other prodrug analogs were effective treatments for experimental murine coccidioidomycosis caused by *C. immitis*. To be sure that the drugs were acting on the tissue stages of this dimorphic fungus and not the arthroconidia used to infect the mice (28), treatment was delayed until 7 days after infection. Thus, the infection more closely mimicked treatment of coccidioidomycosis pneumonia, as would be seen in clinical practice. The appearance of the organisms in the APX prodrug-treated mice at the end of therapy, as determined by histological analyses of lung tissue sections, was that of immature spherules, suggesting that was their stage of development when treatment began and that the APX drugs prevented further maturation.

We assessed two all-oral-treatment regimens that led to similar reductions in fungal burden. Mice were treated either with 50 mg/kg BID of APX001, or they were pretreated with 50 mg/kg of the pan-CYP450 inhibitor ABT 2 h prior to administering the APX prodrugs at 26 mg/kg once daily. ABT prolonged the half-life and increased the exposure of the APX active moieties by 8.6- to 15-fold, so that once-daily dosing with ABT achieved similar or better therapeutic benefits than multiple higher doses of the APX molecules without ABT. This is consistent with *in vivo* efficacy being a function of drug exposure, as has been observed for APX001 and its analogs in other infection models (17, 18, 22). The oral 26-mg/kg QD treatment regimen reduced colony counts, as well as the twice-daily oral treatment with 50 mg/kg fluconazole, given for the same duration. Although fluconazole is not the most active triazole against the mold form of *Coccidioides*, it is considered first-line therapy for coccidioidomycosis (7) and is easy to administer orally in mice because it is water soluble (29).

When we compared the ability of fluconazole and the APX drugs to prolong survival after the end of therapy, we found that the two Gwt1 inhibitor prodrugs, APX001 and APX2097, were superior to fluconazole (*P* < 0.01) in that they prolonged survival for many days after treatment ended (Fig. 7). Although the functions of GPI-linked proteins in *Coccidioides* are still unknown, the antifungal activity of the Gwt1 inhibitors both *in vitro* and *in vivo* implies that they are of vital importance for both the hyphal and the spherule stages of the fungus. The more prolonged survival after treatment was stopped may be due to a longer postantifungal effect of the Gwt1 inhibitors (30), better immune system recognition due to a loss of mannoproteins (13), or other factors. Further work is needed to determine the factors that result in the persistent activity of the Gwt1 inhibitors after treatment ended.

The APX drugs were also tested against the mold form of the fungus *in vitro*. One of the difficulties in evaluating the activity of compounds *in vitro* against dimorphic *Coccidioides* spp. is the lack of standardized CLSI methodology (31). Perhaps of more significance, *in vitro* testing is done against arthroconidia that develop into hyphae under the conditions of the assay, but hyphae are not the pathogenic form of the fungus. We used a broth microdilution methodology similar to the CLSI standard method for determining MEC endpoints (31), and we found the APX drugs to be highly active against the hyphal form of both species of *Coccidioides*. The MEC endpoint has been shown to be a reliable and reproducible method for evaluation of the activity of APX001A (formerly E1210) (23, 24) and the echinocandins (25). A caveat about the significance of MEC *in vitro* results is that the ability to prevent hyphal growth may not be directly relevant to treating infections that are due to spherules. Although one would like to test activity against spherules, since they reproduce by circumferential growth and sequential septation within the spherule (28), monitoring the effect of antifungal drugs on this stage by ordinary microscopy or changes in turbidity *in vitro* is not feasible. Therefore, we tested the drug in an *in vivo* model, and preliminary morphological evidence suggests that APX001A and its analogs also inhibit the growth and maturation of spherules.

Previous susceptibility testing of *Coccidioides* has been performed by broth macrodilution according to methods described in CLSI M38-A3, with MIC values read as the lowest concentration that resulted in ≥80% inhibition of growth versus the no-drug control (26). Using this methodology, a recent study evaluated 377 *Coccidioides* clinical isolates and determined that the posaconazole MIC_90_ was 0.25 μg/ml. These data are similar to the results of the present study where a smaller collection of 10 strains was evaluated using a broth microdilution assay (reading 100% inhibition endpoint) and a posaconazole MIC_90_ of 0.125 μg/ml was observed. Likewise, the previous study showed that the MIC_90_ for fluconazole was 16 μg/ml, with 37% of clinical isolates exhibiting fluconazole MICs of ≥16 μg/ml and 3.8% with MICs of ≥64 μg/ml (9). In the present study, we also observed a fluconazole MIC_90_ of >16 μg/ml (Table 1). Although fluconazole is the most commonly used antifungal agent for *Coccidioides* infections, the use of other agents with lower MIC values, such as the newer triazoles, or Gwt1 inhibitor prodrugs, such as APX001, may be better alternative treatment options for coccidioidomycosis (9).

APX001 is a first-in-class, broad-spectrum antifungal agent that is currently in clinical development for the treatment of life-threatening invasive fungal infections. APX001 has been shown to be effective in mouse models of Candida albicans (18, 22, 32), Candida auris (33), and Cryptococcus neoformans (17) infections, as well as *Aspergillus* and *Fusarium* infections (16). In addition to increased survival, a reduction in the colony counts of fungi in the lungs, kidneys, and brain tissues of infected mice has been observed, consistent with ^14^C-APX001 studies that demonstrated wide tissue distribution in rats and monkeys, especially in tissues associated with invasive fungal infections (34). Notably, treatment with APX001 lead to a significant reduction in brain CFU in both a rabbit model of hematogenous C. albicans meningoencephalitis (35) and a mouse disseminated C. auris model (33). CFU in brain were also examined in this study, and the APX001-treated group resulted in sterilization of the brain in all animals. However, the untreated control group demonstrated low CFU counts (<5 CFU/g of tissue) and thus, although statistical significance was reached (*P* = 0.0002), the low numbers make it difficult to assess biological significance.

We demonstrate here that APX001A, the active moiety of APX001, has good *in vitro* activity against the mold form of *Coccidioides*, with a MEC_90_ of 0.008 μg/ml. Two additional Gwt1 inhibitor analogs, APX2020 and APX2041, demonstrated 2- to 4-fold improved activity versus APX001A with MEC_90_ values of 0.004 and 0.002 μg/ml, respectively, against a panel of C. immitis and C. posadasii strains (Table 2). These values compare favorably with posaconazole (MIC_90_ of 0.125 μg/ml), one of the triazoles that is used clinically for the treatment of coccidioidomycosis (36).

In summary, we found that APX001A and its analogs were highly active *in vitro* against both species of *Coccidioides* and that the oral administration of the corresponding prodrugs was an effective treatment for pulmonary coccidioidomycosis and prevented systemic spread in a genetically susceptible mouse strain. The demonstrated efficacy against *Coccidioides*, as well as previous studies of efficacy against other yeasts and molds, provides support that APX001 is a promising new broad-spectrum antifungal agent worthy of continued investigation.

## MATERIALS AND METHODS

### Isolates tested and organism handling.

All isolates tested were originally clinical isolates. However, C. immitis RS, C. posadasii Silvera, and C. posadasii C735 have been passaged for years in different laboratories. We also collected clinical isolates from cases diagnosed in San Diego over the 24 months prior to the *in vitro* testing (Table 2). Standard biosafety level 3 (BSL3) safety precautions were followed for all *in vitro* work.

### Arthroconidia preparation.

Arthroconidia were prepared as previously described (37). *Coccidioides* colonies were grown on 2× glucose-yeast extract (GYE) agar. The plates were incubated at 30°C until the mycelia covered the surface of the agar. Arthroconidia were harvested from the plate after 4 to 5 weeks of incubation at 25°C by adding 25 ml of saline. The plate was gently scraped using cell scraper, and the fluid was transferred to a 50-ml tube that was then vigorously mixed for 10 s and centrifuged at 3,000 rpm for 10 min at 4°C. The supernatant containing floating mycelia was discarded. The pellet containing arthroconidia was resuspended in saline and passed through three layers of Miracloth (Calbiochem) to filter out mycelia. The strained suspension was centrifuged again and resuspended in saline, and the arthroconidia were quantitated by counting them under a microscope using a hemocytometer. The viability is determined by dilution plating and counting the CFU on GYE agar.

### Reagents.

APX001 is the prodrug of APX001A, APX2097 is the prodrug of APX2020, APX2104 is the prodrug of APX2041, and APX2096 is the prodrug of APX2039 (Amplyx Pharmaceuticals, San Diego, CA) (Fig. 1). Posaconazole and fluconazole solutions were pharmacy grade.

### *In vitro* susceptibility testing.

Drug susceptibility tests were performed using a broth microdilution method according to the Clinical and Laboratory Standard Institute (CSLI) M38-A2 (26). The assay was conducted in RPMI 1640 medium (Sigma) containing 0.165 M morpholinepropanesulfonic acid (MOPS; Sigma) at pH 7.0. Twofold serial dilutions of the drug were made in RPMI 1640 from the highest concentration of 16 μg/ml to the lowest concentration of 0.016 ng/ml. Arthroconidia were diluted in RPMI 1640 medium. Then, 1 μl of the spore suspension was added to 99 μl of drug in one well of a 96-well U-bottom sterile plate (Corning) to a final concentration of 5 × 10^4^/ml. A control well was set up with dimethyl sulfoxide only. Each dilution of the drugs was tested in duplicate, and the plates were incubated at 37°C for 2 to 3 days.

The plates were visually scored using a magnifying mirror to determine the MIC (100% inhibition). The MEC scores were determine by examining each well for growth using an inverted microscope. The MEC endpoint was the lowest drug concentration that uniformly shortened hypha formation. Two independent observers read each well. If there was more than a one-dilution difference in interpretation, a third observer was used.

### Mice.

C57BL/6J (B6) female mice were purchased from Jackson Laboratory at 8 weeks of age and infected 1 week after arrival.

### Infections and treatment.

Standard BSL3 precautions were followed for all *in vivo* work. Mice were infected intranasally as previously described and housed in cages inside a HEPA-filtered glove box that was contained inside a biological safety hood (38). Briefly, the animals were anesthetized with a mixture of ketamine and xylazine, and then ∼200 spores (arthroconidia), suspended in 20 μl of sterile saline, were slowly dropped into their nares. After the mice recovered from the anesthesia, they were placed three or four per cage in a HEPA-filtered glove box inside our BSL3 facility and allowed free access to food and water. Treatment by oral gavage while the mice were inside the biological safety hood was initiated 7 days postinfection and continued for 5 days. Fluconazole was administered orally as an aqueous solution at a dose of 25 mg/kg twice daily, and APX001 was diluted in 5% glucose and dosed orally at 50 mg/kg twice a day for 10 days in the first experiment. Mice were sacrificed 1 day after the last dose. In all subsequent experiments, treatment was initiated 7 days of infection using a regimen of 50 mg/kg of ABT by oral gavage, followed 2 h later by oral gavage with 26 mg/kg of an APX prodrug. Treatments continued for 5 days with control mice receiving 50 mg/kg ABT, followed by buffer. One day after treatment ended (day 13 postinfection) mice were sacrificed for quantitative culture of lungs and spleens, as previously described (38). The infection and quantitation of CFU with APX001 was repeated three times, with some minor variations in dosing but similar outcomes. Fluconazole was only tested once, but the results were consistent with previously published findings (39).

### Histology.

On the last day of treatment, mouse lungs were removed *en bloc* and then inflated through the trachea with glutaraldehyde. The lungs were then fixed overnight in glutaraldehyde and stained with periodic acid-Schiff (PAS) stain according to standard methods. PAS stains polysaccharides.

### Statistics.

Colony counts were log_10_ transformed and geometric mean CFU/organ ± the standard errors of the mean (SEM) were calculated; two groups were compared using an unpaired *t* test (Prism, v7.01; GraphPad, San Diego, CA). If there were greater than two groups, the differences in the means of the treated and control groups were compared using Dunnett’s analysis of variance (ANOVA) test. Kaplan-Meier survival curves were compared by log rank testing (Prism, v7.01). A *P* value of ≤0.05 was considered statistically significant.
